# Integrated metabolomics and transcriptomics analysis unveils key metabolic pathways and potential biomarkers in clear cell renal cell carcinoma

**DOI:** 10.1007/s12672-026-04612-1

**Published:** 2026-04-14

**Authors:** Xiaoguang Zhou, Kunpeng Zhang, Zhendong Wang, Lulu Yang, Yongzhuo Zhang, Zhichao Xue

**Affiliations:** 1 Department of Urology, Beijing Chao-Yang Hospital, Capital Medical University, Beijing, 100020 People’s Republic of China; 2 Technology Innovation Center of Mass Spectrometry for State Market Regulation, Center for Advanced Measurement Science, National Institute of Metrology, Beijing, 100029 People’s Republic of China; 3 School of Life Science and Engineering, Lanzhou University of Technology, Lanzhou, 730050 People’s Republic of China; 4 Center for Advanced Measurement Science, National Institute of Metrology, Beijing, 100029 People’s Republic of China

**Keywords:** Renal clear cell carcinoma, Transcriptomics, Metabolomics, Biomarker

## Abstract

Clear cell renal cell carcinoma (ccRCC) is the most prevalent histological subtype of renal cancer, with both incidence and mortality rates on the rise in recent years. This underscores the urgent need for precise diagnostic tools, effective monitoring, and improved management strategies. Advances in tumor sequencing have significantly enhanced our understanding of the molecular biology of ccRCC, leading to the identification of several potential genomic biomarkers. However, no widely accepted tissue or blood biomarker for ccRCC has been incorporated into routine clinical practice. In this study, we integrated transcriptomics and metabolomics analyses using paired cancerous and non-cancerous tissue samples from five patients who underwent radical nephrectomy. Our goal was to systematically investigate the molecular mechanisms underlying ccRCC and identify potential therapeutic targets. We identified ten shared KEGG metabolic pathways between the transcriptome and metabolome, with purine metabolism and glycerophospholipid metabolism emerging as particularly relevant to tumorigenesis and disease progression. Network analysis revealed significant correlations between specific metabolites (e.g., guanosine triphosphate and L-malic acid) and key genes (e.g., MET, SDHC, FH, and SDHB) within the molecular network of ccRCC. Additionally, we identified key enzymes in phospholipid metabolism, such as AK, LPCAT, and LPEAT, as potential therapeutic targets. Inhibiting these pathways could effectively disrupt tumor membrane formation and signal transduction. Our findings not only deepen the understanding of the molecular mechanisms driving ccRCC but also provide valuable insights for early diagnosis, prognosis evaluation, and the development of novel therapeutic strategies. Future work should focus on validating these potential biomarkers and therapeutic targets in larger cohorts and exploring their clinical utility.

**Supplementary Information:**

The online version contains supplementary material available at 10.1007/s12672-026-04612-1.

## Introduction

Renal cell carcinoma (RCC) is one of the most common malignant tumors of the urinary system [1], ranking as the 9th most common cancer in men and the 14th in women globally [2, 3]. Unlike many other cancer types, the incidence of kidney cancer has been increasing annually [4]. In 2020, there were approximately 431,000 new cases of kidney cancer worldwide, with 271,000 cases in men and 160,000 cases in women; kidney cancer caused 179,000 deaths, with 115,000 in men and 64,000 in women [5]. Among the various subtypes of RCC, clear cell renal cell carcinoma (ccRCC) is the most common, accounting for approximately 75% to 80% of all kidney cancer cases, and is known for its high invasiveness [6–8]. Although early detection and surgical resection or ablation strategies can lead to successful treatment [9], ccRCC is often asymptomatic and undetected until it reaches an advanced stage, with metastasis in some cases. The 5-year survival rate for patients with advanced or metastatic kidney cancer is less than 20% in 30% of cases [10]. The development of ccRCC involves complex molecular mechanisms, including the aberrant activation of multiple signaling pathways and oncogenes, which contribute to tumor heterogeneity and resistance to treatment [11–13]. Therefore, research into identifying ccRCC-specific biomarkers and the metabolic pathways involved is of great significance, as these studies are crucial for the early diagnosis and effective treatment of ccRCC.

In recent years, the development of transcriptomics and metabolomics has provided new perspectives for studying ccRCC. Transcriptomics, by analyzing the RNA composition of cells or tissues, offers a panoramic view of gene expression patterns. In ccRCC research, transcriptomics has successfully identified specific gene expression patterns related to tumor development, invasion, and prognosis. Junyi Hu et al., through transcriptomic data analysis, identified 15 major cell types and 39 cell subpopulations derived from either tumor or non-tumor tissues. They confirmed that T cell exhaustion is a key factor in the immunosuppressive characteristics of ccRCC tissues, which is significantly associated with poor prognosis. They also discovered that abnormal metabolic patterns occur not only in cancer cells but also in tumor-infiltrating stromal cells [14]. Fuzhong Liu et al., by analyzing transcriptomic sequencing data from 6 ccRCC and 6 normal kidney tissues, demonstrated that the transcription factor POU5F1 can upregulate the expression of SPP1. SPP1 binds to integrin receptors on target cell surfaces and promotes ccRCC development and progression by activating potential signaling mechanisms such as ILK and JAK/STAT [15]. Zhenyuan et al. revealed the regulatory features of different tumor cell subtypes in ccRCC and identified two long non-coding RNAs (RP11-661C8.2 and CTB-164N12.1) that promote ccRCC invasion and migration [16]. Metabolites are the substances, intermediates, and products of various biochemical reactions and serve as the best reflection of physiological and pathological processes occurring in the human organism [17]. Metabolomics, by analyzing changes in cellular and small molecule metabolites, provides direct information about the metabolic state of tumor cells. This information helps uncover molecular subtypes of tumors and provides potential targets for personalized treatment. Hakimi AA et al., through metabolomic analysis, found that ccRCC is characterized by extensive alterations in central carbon metabolism, one-carbon metabolism, and antioxidant responses. Tumor progression and metastasis are associated with increased metabolites in the glutathione and cysteine/methionine metabolic pathways [18]. Teng R et al., using isotope tracing techniques in combination with metabolomics and proteomics, revealed that HSP60 knockdown drives metabolic reprogramming in ccRCC, promoting tumor progression and enhancing mitochondrial-dependent biosynthesis [19]. The studies on transcriptomics and metabolomics have not only deepened our understanding of the molecular mechanisms underlying ccRCC but also provided important information for the development of new therapeutic strategies and prognostic evaluations. These studies also offer new insights into identifying biomarkers and key pathways for ccRCC progression.

The integrated analysis of metabolomics and transcriptomics data helps identify genes and metabolic pathways that may play key roles in tumor initiation and progression, while also revealing the connection between gene expression changes and metabolite level alterations. This approach provides a more comprehensive perspective for understanding the metabolic regulation and molecular mechanisms of ccRCC, identifying potential therapeutic targets, and offering clues for the development of new treatment strategies. The aim of this study is to use transcriptomics and metabolomics technologies to conduct a comprehensive analysis of ccRCC samples by identifying differentially expressed genes and differentially abundant metabolites in ccRCC samples. The goal is to construct a gene-metabolite network to uncover key regulatory nodes in tumor progression and explore these molecules as potential biomarkers for early diagnosis and prognostic evaluation of ccRCC, as well as important signaling pathways involved in the disease.

## Materials and methods

### Materials and sample preparation

In total, we collected tissue specimens from 5 patients who underwent radical nephrectomy, including tumor tissue and normal renal cortex tissue (Table 1). The pathology of ccRCC and normal kidney tissue was validated by at least two pathologists. The cohort included 2 male and 3 female patients, with a median age of 71 years (mean ± standard deviation, 68.2 ± 9.5 years; range, 52–81 years). Tissue samples were collected in sterile containers, which were stored at 4 °C until transported to the laboratory for processing. The samples were frozen at − 80 °C within 5 h after collection for further analysis. The collection of samples used in this study was approved by the Ethics Committee of Beijing Chaoyang Hospital, Capital Medical University. (Acceptance Number: 2020–12-22–2).

**Table 1:** Sample information table

Sample No.	Code	Gender	Age	BMI	Grade(WHO/ISUP)	Tumor side
2023C104-1	1,769,821	Female	66	24.03	1–2	Left
2023C104-2	1,611,074	Male	71	24.8	1–2	Right
2023C104-3	1,771,580	Female	81	23.5	3	Right
2023C104-4	1,774,364	Female	52	21.5	2	Left
2023C104-5	1,770,124	Male	71	22.31	2–3	Left

### RNA-seq and data analysis

Total RNA was extracted using TRIzol reagent according to the manufacturer's instructions. The purity and quantification of RNA were assessed using the NanoDrop 2000 spectrophotometer (Thermo Scientific, Wilmington, DE, USA), and the integrity of RNA was evaluated using the Agilent 2100 Bioanalyzer (Agilent Technologies, CA, USA). mRNA was enriched with oligo (dT)-attached magnetic beads and then the fragmentation buffer was exploited to randomly fragment the mRNA into short fragments. Using these cleaved RNA fragments as a template, the first cDNA strand was synthesized by random hexamers, then the second cDNA strand was synthesized by adding buffer, dNTPs, RNaseH. The purified double-stranded cDNA was then end-repaired, A-tailed, and connected to sequencing junctions, followed by fragment size selection with AMPure XP beads; finally, the cDNA library was enriched by PCR. The library was quantified using Qubit and real-time fluorescent quantitative PCR, and the fragment size distribution was detected using the Bioanalyzer. Sequencing was performed with the Illumina novaseq 6000 platform (Illumina, San Diego, CA, USA).

**Fig. 1:**
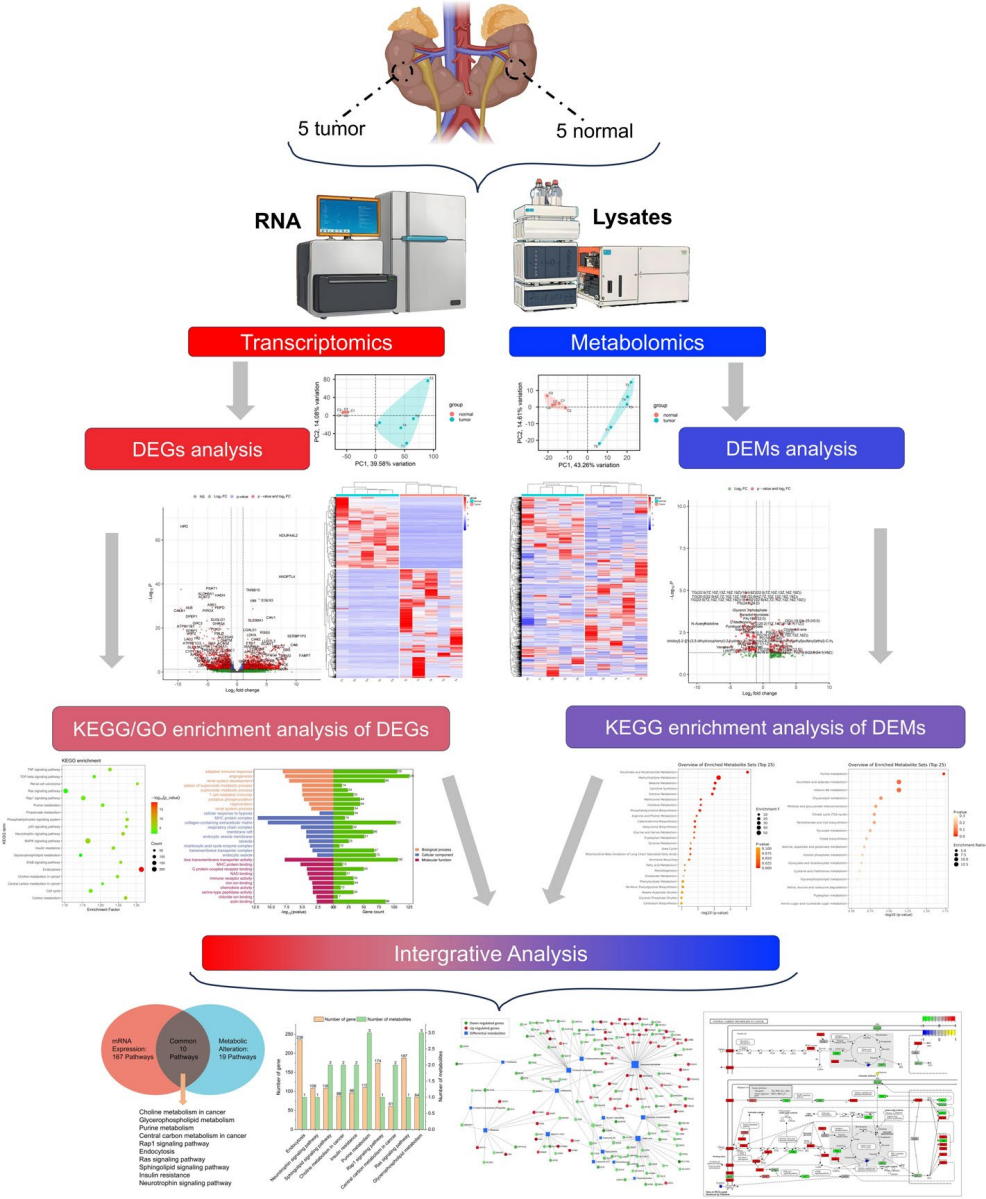
Integrated metabolomic and transcriptomic strategy. We considered biopsies of ccRCC tumors (N = 5) and adjacent normal tissues (N = 5), and generated RNA‐sequencing and metabolomics profiles. The transcriptomic and metabolomic data were integrated by identifying coordinated changes between tumor and matched normal samples via either differential expression, and enriched pathways were inferred. ccRCC, Clear cell renal cell carcinoma

Transcriptome sequencing data were filtered with fastp software to remove adapter reads, low-quality reads, and obtain clean reads. At the same time, Q30 and GC content calculations were performed on the clean data. All downstream analyses were based on high-quality clean data. The clean reads were mapped to the reference genome using HISAT2 (v2.2.1). Using the software FeatureCounts (v2.0.1) for expression quantification to obtain read counts. Then, the transcript per million (TPM) of each gene was calculated based on the length of the gene and reads count mapped to this gene. The differential expression analysis of two groups was performed using the DESeq2 (v1.42.1). The resulting P values were adjusted using the Benjamini–Hochberg approach for controlling the false discovery rate (FDR). Genes with FDR ≤ 0.05 and |log2Fold Change|≥ 1 were considered DEGs. The enrichment analysis of differentially expressed genes annotated to the GO database between sample groups is performed using the ClusterProfiler (v4.10.1) package. At the same time, the enrichment of differentially expressed genes in KEGG pathways is analyzed to understand their degree of enrichment in biological pathways (Fig. 1). Visualization of RNA-seq data is achieved through the R package and the website (www.bioinformatics.com.cn).

### Non-targeted metabolome determination and analysis

The tissue samples were thawed on ice and washed three times with PBS. Magnetic beads were added to each sample tube, and the samples were homogenized using a high-throughput tissue grinder at a speed of 25 Hz for 5 min. After homogenization, 1.5 mL of a pre-chilled solution of chloroform and methanol (v:v, 3.1) at − 80 °C was added to each sample tube and mixed well. The mixture was then centrifuged at 14,000 *g* for 10 min using a centrifuge at 4 °C. The supernatant was transferred to clean centrifuge tubes and allowed to air dry in a fume hood. After the samples were dried, 120 μL of a methanol solution (v:v, 1:1) was added to re-dissolve the samples, followed by thorough mixing, and the solution was prepared for further analysis.

For the analysis of polar molecules, both positive and negative ion modes were used. In positive ion mode, an Atlantis HILIC column (3 μm, 2.1 × 150 mm) was employed. The mobile phase A consisted of a mixture of water, 10 mM ammonium formate, and 0.1% formic acid, while mobile phase B was a mixture of acetonitrile and 0.1% formic acid. The method program lasted for 15 min with a constant flow rate of 0.25 mL/min. From 0 to 1 min, mobile phase B was 95%, then reduced to 40% between 1 and 9 min and maintained for 4 min. Afterward, the percentage of mobile phase B was increased to 95% in 2 min, completing the program. For negative ion mode, an XBridge BEH Amide column (2.5 μm, 2.1 × 150 mm) was used. The mobile phase A was a mixture of 95% water, 5% acetonitrile, 10 mM ammonium hydroxide, and 10 mM ammonium acetate, while mobile phase B was composed of 5% water, 95% acetonitrile, 10 mM ammonium hydroxide, and 10 mM ammonium acetate. The method program was conducted at a flow rate of 0.30 mL/min. From 0 to 1 min, mobile phase B was 100%, then decreased to 75% from 1 to 17 min and held for 4 min. The percentage of mobile phase B was increased to 100% in 1 min and maintained for 3 min.

For the analysis of non-polar molecules, positive ion mode was used with a Kinetex C18 column (2.6 μm, 150 × 2.1 mm). The method program was conducted at a flow rate of 0.25 mL/min. From 0 to 1 min, mobile phase B was 0%, then increased to 50% from 1 to 6 min. From 6 to 30 min, mobile phase B was further increased to 100% and maintained for 2 min. Afterward, mobile phase B was decreased to 0% within 6 s and held for 3.9 min (see Fig. 1).

Data analysis and visualization were performed using the software R and the website [MetaboAnalyst] (https://www.metaboanalyst.ca/).

## Results

### Analysis of differentially expressed genes

The transcriptomic sequencing results of 5 normal kidney tissue samples and 5 tumor tissue samples yielded a total of 65.66 G of raw data. After filtering, 63.85 G of clean data was obtained. The alignment rate on the reference genome ranged from 94.40 to 96.63%, with a unique alignment rate ranging from 87.71 to 91.53%. The percentage of Q30 bases in each sample ranged from 91.46 to 95.45%, and the GC content ranged from 45.16 to 46.38% (see Table S1). These results indicate that the sampling for the experiment was reasonable, and the RNA-seq data quality is reliable.

**Fig. 2:**
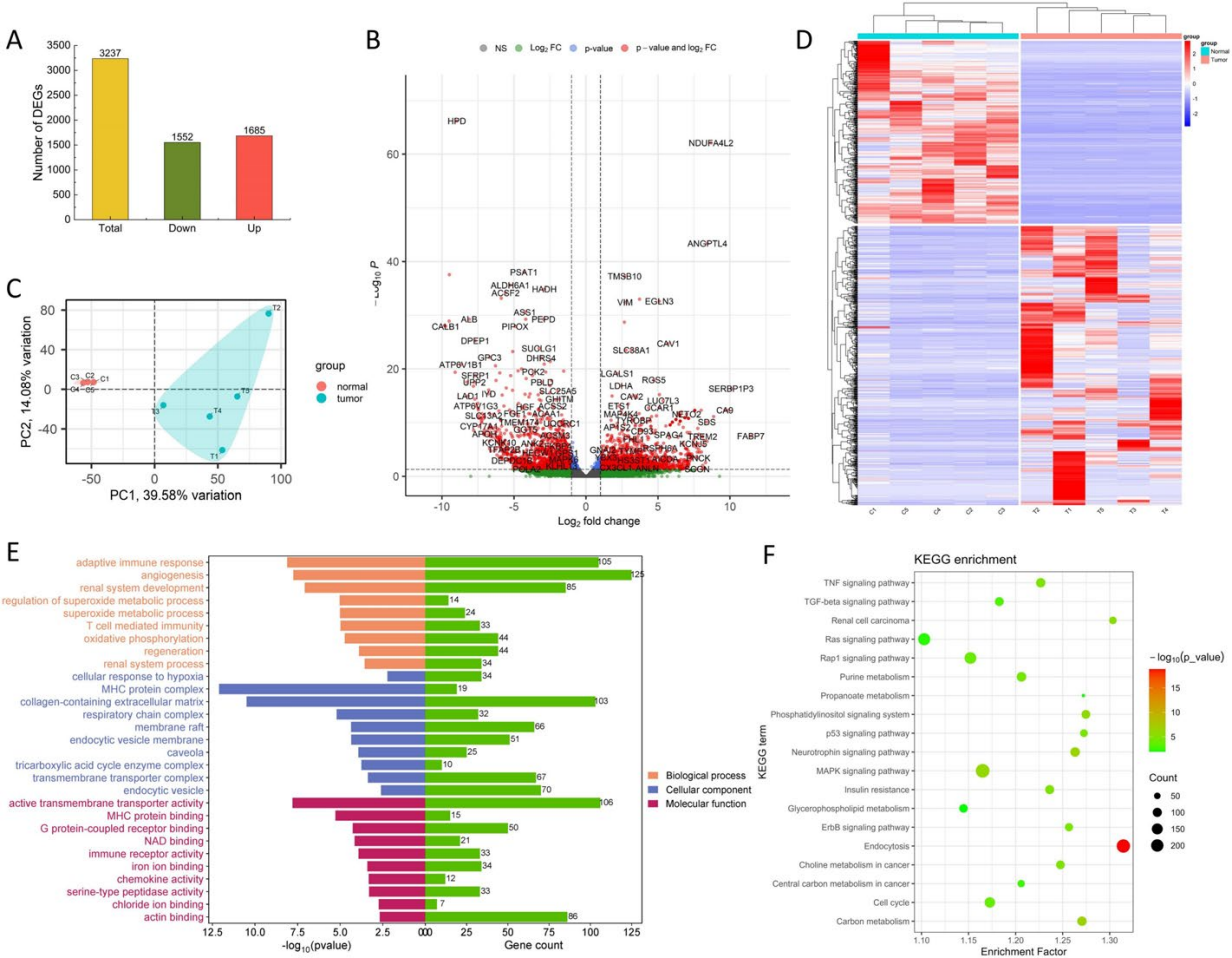
Transcriptomics analysis of ccRCC. **a** Heatmap and hierarchical clustering showing DEGs. **b** PCA analysis of transcriptomics data from tumor and normal groups. **c** The number of DEGs. **d** Volcano plots of the DEGs. **e** GO pathway enrichment plots of transcriptomic data. **f** KEGG pathway enrichment plots of transcriptomic data. PCA, Principal component analysis. DEGs, Differentially expressed genes. GO, Gene ontology. KEGG, Kyoto Encyclopedia of Genes and Genomes

The RNA-seq results were aligned to the reference genome, and an analysis of the kidney tumor tissue samples and normal tissue samples was performed, identifying a total of 18,242 genes (Table S2). Clustering analysis revealed significant differences in gene expression patterns between the tumor and normal tissue samples, which were clearly shown in the clustering heatmap (Fig. 2a), where red and blue represent upregulated and downregulated genes, respectively. Additionally, principal component analysis (PCA) was conducted on the genes of the tumor and normal tissue samples. Principal component 1 (PC1) and principal component 2 (PC2) explained 39.58% and 14.08% of the variance, respectively. The PCA score plot further demonstrated that the samples within each group clustered tightly, indicating good reproducibility (Fig. 2b). Both the clustering heatmap and the PCA score plot showed clear separation between tumor and normal tissue samples, with each group forming an independent cluster, further supporting the previous results. Differentially expressed genes between tumor and normal tissue samples were selected using the criteria of |log2Fold Change|> 1 and a false discovery rate (FDR) < 0.05. A total of 3237 differentially expressed genes were identified, of which 1,685 were upregulated and 1552 were downregulated (Fig. 2c, d).

GO enrichment analysis of the differentially expressed genes revealed that, in the biological process (BP), molecular function (MF), and cellular component (CC) categories, kidney tumor tissue samples were annotated with 259, 58, and 20 GO terms, respectively, compared to normal tissue samples (Table S2). The GO terms related to kidney cancer in each category were visualized (Fig. 2e). The results showed that the differentially expressed genes in tumor tissue samples, compared to normal tissue samples, were primarily enriched in biological processes such as *adaptive immune response*, *angiogenesis*, *renal system development*, *regulation of superoxide metabolic process*, *superoxide metabolic process*, *T cell mediated immunity*, *oxidative phosphorylation*, *regeneration*, and *renal system process*. In molecular functions, the genes were mainly enriched in *active transmembrane transporter activity*, *MHC protein binding*, *G protein-coupled receptor binding*, *NAD binding*, *immune receptor activity*, *iron ion binding*, *chemokine activity*, *serine-type peptidase activity*, *chloride ion binding*, and *actin binding*. In cellular components, the significant terms included *cellular response to hypoxia*, *MHC protein complex*, *collagen-containing extracellular matrix*, *respiratory chain complex*, *membrane raft*, *endocytic vesicle membrane*, *caveola*, *tricarboxylic acid cycle enzyme complex*, *transmembrane transporter complex*, and *endocytic vesicle*. Among these, four GO terms—*adaptive immune response*, *angiogenesis*, *active transmembrane transporter activity*, and *collagen-containing extracellular matrix*—contained the most differentially expressed genes, with 105, 125, 106, and 103 genes, respectively.

Through KEGG pathway enrichment analysis, a total of 177 related pathways were identified (Table S3), and 20 of the pathways most relevant to kidney cancer were visualized (Fig. 2f). The KEGG enrichment analysis results indicated that, compared to normal tissue samples, the differentially expressed genes in tumor tissue samples were primarily enriched in the following signaling pathways: *TNF signaling pathway*, *Ras signaling pathway*, *Rap1 signaling pathway*, *Purine metabolism*, *MAPK signaling pathway*, *Endocytosis*, *Choline metabolism in cancer*, *Cell cycle*, and *Carbon metabolism*, among others.

### Analysis of differential accumulation metabolites

**Fig. 3:**
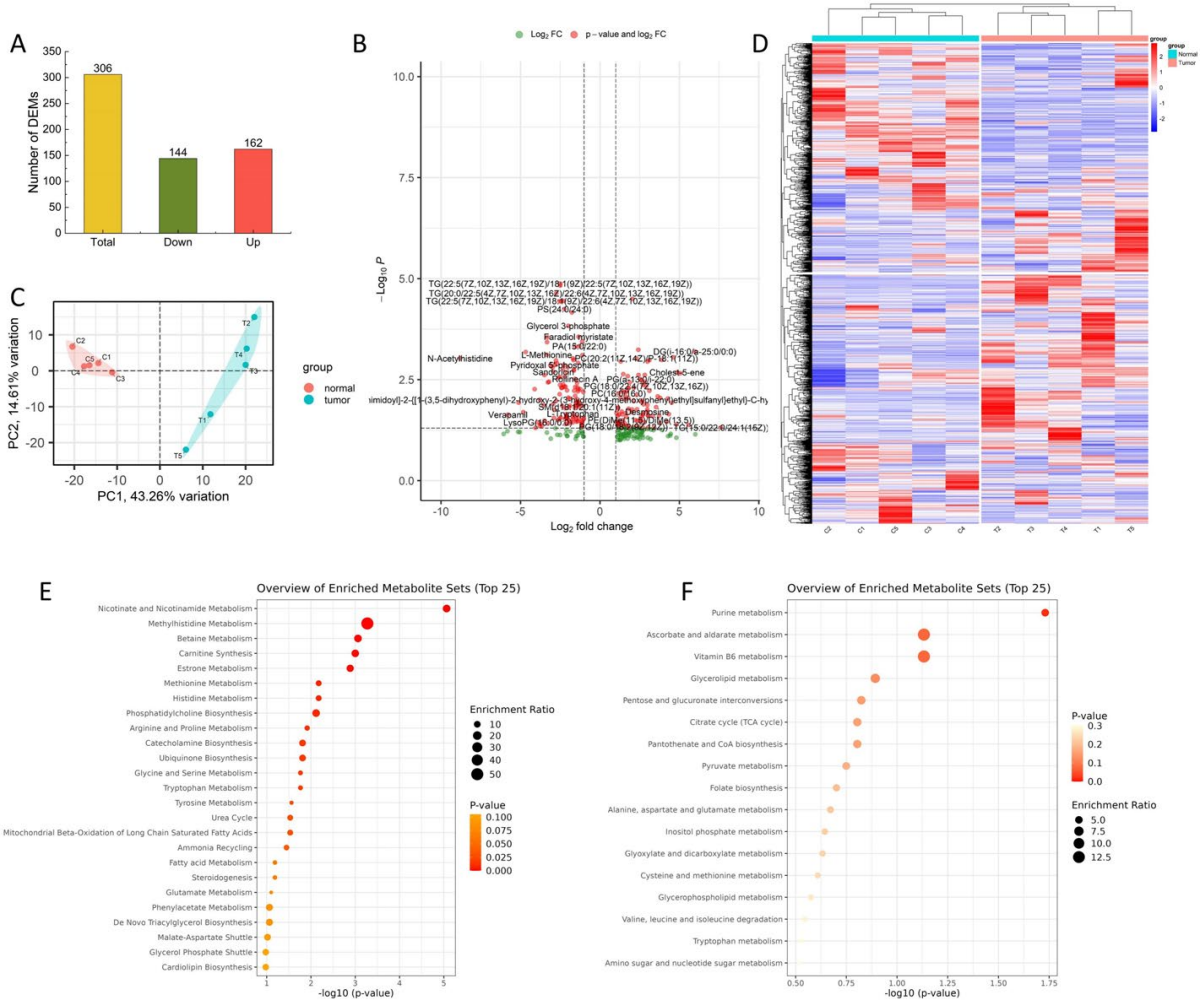
Metabolomics analysis of ccRCC. **a** Heatmap and hierarchical clustering showing DAMs. **B** PCA analysis of metabolomics data from tumor and normal groups. **c** The number of DAMs. **d** Volcano plots of the DAMs. **e** KEGG pathway enrichment plots of up-regulated metabolites. **f** KEGG pathway enrichment plots of down-regulated metabolites. DAMs, Differentially accumulated metabolites. PCA, Principal component analysis. KEGG, Kyoto Encyclopedia of Genes and Genomes

The metabolomic data analysis identified a total of 1277 metabolites (Table S5). Clustering analysis showed distinct differences in the metabolite profiles between tumor and normal tissue samples, which were clearly illustrated in the clustering heatmap (Fig. 3a), where red and blue represent upregulated and downregulated metabolites, respectively. Principal component analysis (PCA) was performed on the metabolites of tumor and normal tissue samples. Principal component 1 (PC1) and principal component 2 (PC2) accounted for 43.26% and 14.6% of the variance, respectively. The PCA score plot further showed tight clustering within each sample group, indicating good reproducibility (Fig. 3b). Both the clustering heatmap and the PCA score plot demonstrated clear separation between tumor and normal tissue samples, with each group forming independent clusters, further supporting the aforementioned results. Differential metabolites between tumor and normal tissue samples were selected using the criteria of |log2Fold Change|≥ 1 and Variable Importance in Projection (VIP) ≥ 1. A total of 306 differential metabolites were identified, including 162 upregulated metabolites and 144 downregulated metabolites (Fig. 3c, d).

**Fig. 4:**
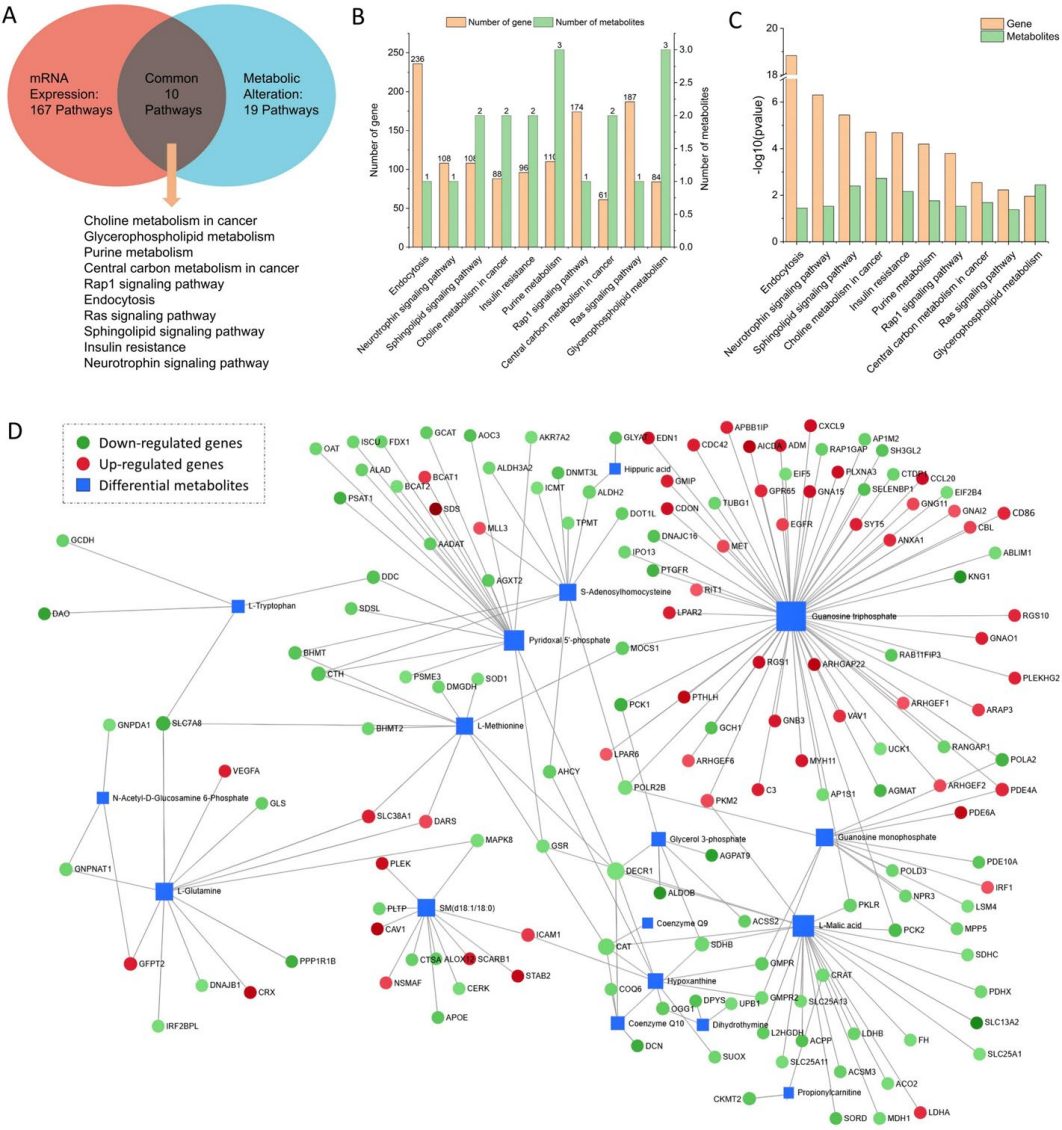
Profiles of integrated analysis of DEGs and DAMs. **a** Venn diagram of co-enrichment pathways for DEGs and DAMs. **b** Column diagrams of differential number of co-enrichment pathways for DEGs and DAMs. **c** Column diagrams of differential significance of co-enrichment pathways for DEGs and DAMs. **d** Network diagram of the interaction among DEGs and DAMs. DEGs, Differentially expressed genes. DAMs, Differentially accumulated metabolites

KEGG pathway enrichment analysis was performed for the upregulated and downregulated differential metabolites, and the results were visualized. The upregulated metabolites were enriched in 11 related pathways (Fig. 3e), while the downregulated metabolites were enriched in 21 related pathways (Fig. 4f).The KEGG enrichment analysis results indicated that, compared to normal tissue samples, the differential metabolites in tumor tissue samples were primarily enriched in the following signaling pathways: *Leishmaniasis*, *Glycerophospholipid metabolism*, *Sphingolipid signaling pathway*, *Retrograde endocannabinoid signaling*, *Insulin resistance*, *Choline metabolism in cancer*, *Glycerophospholipid metabolism*, *Retrograde endocannabinoid signaling*, *Riboflavin metabolism*, *Sphingolipid metabolism*, *Mineral absorption*, *Purine metabolism*, and others.

### Combined analysis of transcriptome and metabolome

To further investigate the molecular mechanisms and metabolic pathways driving the development of clear cell renal cell carcinoma (ccRCC), we performed a combined analysis of transcriptomic and metabolomic data. The results revealed that 10 KEGG metabolic pathways were shared between the transcriptome and metabolome. These pathways include *Choline metabolism in cancer*, *Glycerophospholipid metabolism*, *Purine metabolism*, *Central carbon metabolism in cancer*, *Rap1 signaling pathway*, *Endocytosis*, *Ras signaling pathway*, *Sphingolipid signaling pathway*, *Insulin resistance*, and *Neurotrophin signaling pathway* (Fig. 4a). We then visualized the number of differentially expressed genes and differentially abundant metabolites enriched in these 10 pathways, along with their significance (Fig. 4b, c).

**Fig. 5:**
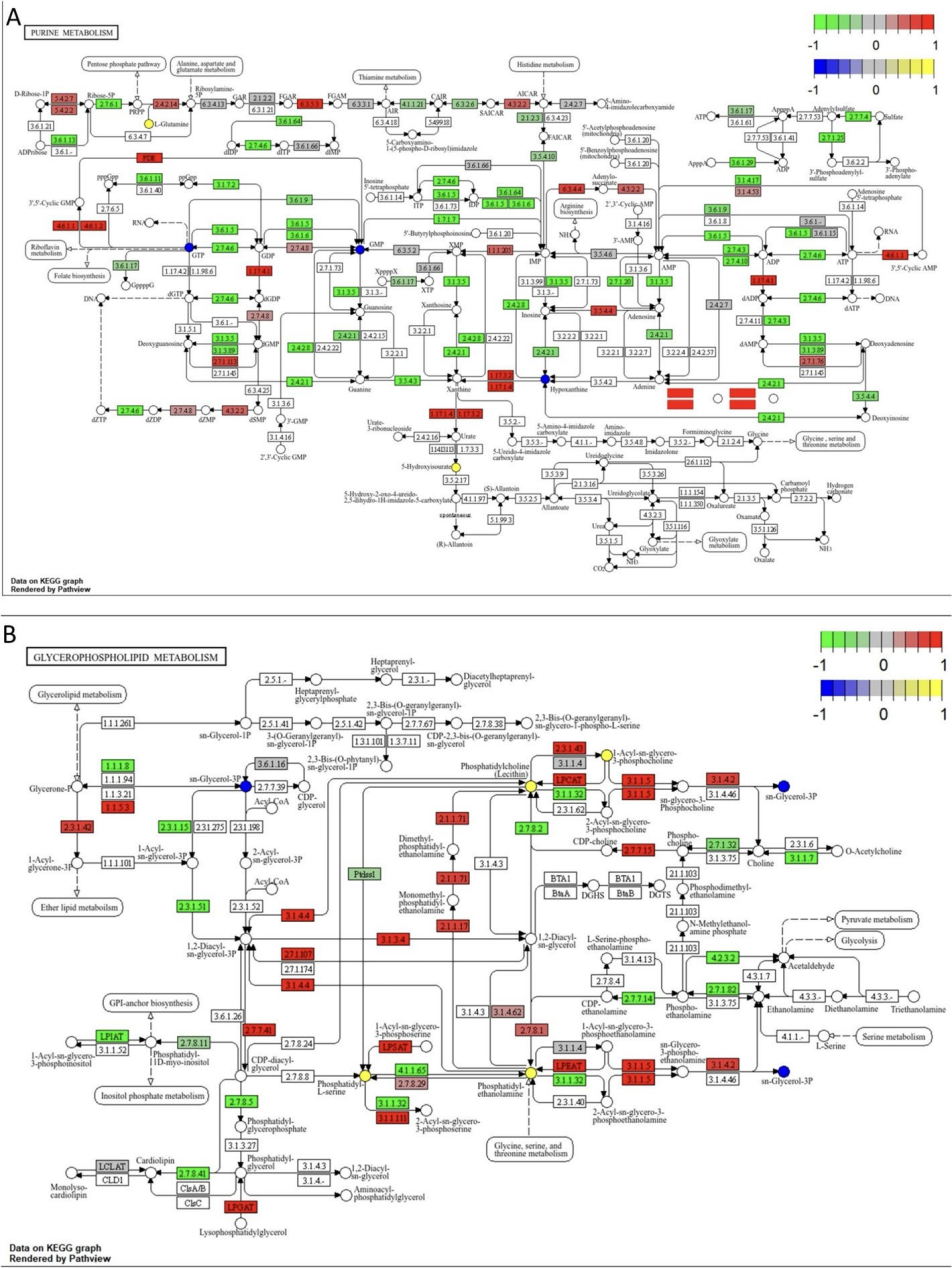
Important pathway map of co-enrichment pathways of DEGs and DAMs. **a** Purine metabolism pathway. **b** Glycerophospholipid metabolism pathway

Meanwhile, we explored how differentially expressed genes influence metabolite changes by combining transcriptomic and metabolomic data, and constructed an interaction network diagram of differentially expressed genes and metabolites (Fig. 4d). From the network, we found that metabolites highly correlated with differentially expressed genes include *Guanosine triphosphate*, *Guanosine monophosphate*, *Glycerol-3-phosphate*, *L-Malic acid*, *S-Adenosylhomocysteine*, *N-Acetyl-D-Glucosamine 6-Phosphate*, *L-Tryptophan*, and *Hypoxanthine*.We then annotated the differentially expressed genes and metabolites onto the KEGG pathway map of the 10 shared pathways and generated a pathway map (Figure SF1). Among these pathways, we specifically highlighted two pathways—*Purine metabolism* and *Glycerophospholipid metabolism*—which contain metabolites highly correlated with differentially expressed genes, such as *Guanosine triphosphate*, *Guanosine monophosphate*, *Glycerol-3-phosphate*, and *Hypoxanthine* (Fig. 5).

From the *Purine metabolism* pathway map (Fig. 5a), we observed significant upregulation of several enzyme genes associated with nucleotide synthesis, such as *Adenylate cyclase* (EC 4.6.1.1), *Phosphoribosylglycinamide formyltransferase* (EC 6.3.5.3), and *Ribonucleoside diphosphate reductase alpha chain* (EC 1.17.4.1). In contrast, multiple enzyme genes related to nucleotide degradation, including *5'-nucleotidase* (EC 3.1.3.5), *Guanosine deaminase* (EC 3.5.4.3), and *Purine nucleoside phosphorylase* (EC 2.4.2.1), were significantly downregulated. The alterations in these key genes suggest a clear imbalance in nucleotide metabolism in ccRCC, which consequently leads to the upregulation of *IMP* (inosine monophosphate, EC 1.1.1.205). IMP can be converted into *AMP* and *GMP*. However, the pathway analysis shows that *GMP* is actually downregulated, which seems counterintuitive at first glance. Upon closer examination, we found that the gene for *Adenosine kinase* (EC 2.7.1.20), which is involved in ATP metabolism, was significantly downregulated. This appears to resolve the paradox, as the downregulation of *Adenosine kinase* could impair the conversion of *IMP* into *AMP* and *GMP*, further contributing to the observed metabolic imbalance.

According to the *Glycerophospholipid metabolism* pathway map (Fig. 5b), we observed significant upregulation of key genes associated with *Phosphatidylcholine (PC)* synthesis, such as *Cholinephosphotransferase* (EC 2.7.7.15) and *Phosphatidylethanolamine/Phosphatidyl-N-methylethanolamine N-methyltransferase* (EC 2.1.1.71). Similarly, key genes involved in the synthesis of *Phosphatidylethanolamine (PE)*, including *Phosphatidic acid cytidylyltransferase* (EC 2.7.7.41) and *Ethanolaminephosphotransferase* (EC 2.7.8.1), were also significantly upregulated in ccRCC tissues.Moreover, the upregulation of *Acidic phospholipase A2* (LPCAT) and *Lysophosphatidylcholine acyltransferase* (LPEAT), along with the related metabolite *Phosphatidylserine*, suggests an active phospholipid remodeling process in cancer tissues. This remodeling could play a crucial role in maintaining membrane fluidity and supporting cancer cell signaling pathways. These findings indicate that altered glycerophospholipid metabolism may contribute to the enhanced membrane dynamics and signaling crucial for ccRCC progression.

## Discussion

In the field of research on clear cell renal cell carcinoma (ccRCC), various methods have been employed by scholars [20–22], including transcriptomics and metabolomics, to gain a deeper understanding of the molecular mechanisms underlying the disease [23–27]. However, reports combining both omics approaches for multi-omics research are relatively few [28]. In this study, by integrating transcriptomic and metabolomic data, we identified 10 common signaling pathways, namely, Choline metabolism in cancer, Glycerophospholipid metabolism, Purine metabolism, Central carbon metabolism in cancer, Rap1 signaling pathway, Endocytosis, Ras signaling pathway, Sphingolipid signaling pathway, Insulin resistance, and Neurotrophin signaling pathway. The alterations in these pathways have been directly or indirectly linked to kidney cancer progression, as supported by previous studies. It is important to emphasize that ccRCC is fundamentally a metabolic disease characterized by profound reprogramming of energy metabolism, including enhanced glycolysis, impaired oxidative phosphorylation, and dysregulated lipid metabolism [29, 30]. In particular, dysregulation of lipid metabolism has been confirmed as a key factor driving renal carcinogenesis [31].

For instance, Glunde K et al. pointed out that aberrant choline metabolism has become one of the most consistent markers of cancer, and modifying the expression or activity of enzymes involved in choline phospholipid metabolism could serve as a novel therapeutic target for cancer treatment. Specifically, phosphatidylcholine-specific phospholipase D (PtdCho-PLD), involved in cell proliferation and oncogenic signaling, is considered a potential therapeutic target, particularly in breast cancer [32]. Bensaad K et al. demonstrated that under hypoxic conditions, HIF-1α-induced lipid droplet (LD) accumulation is critical for tumor cell growth and survival. This suggests that glycerophospholipid metabolism may be altered under hypoxic conditions to support cellular metabolic demands [33]. Furthermore, glycerophospholipids are key components of cell membranes, involved in cell signaling and energy metabolism. Heravi G et al. mentioned in their study that RCC cells contain high levels of triglycerides and cholesterol, predominantly in the form of cholesteryl ester (CE), which may be linked to RCC's resistance to treatment [34]. Singer K et al. found that, compared to corresponding normal renal tissues, the expression of lactate dehydrogenase A (LDHA) and glucose transporter 1 (GLUT-1) was significantly increased at the mRNA level in RCC samples [35]. This suggests that tumor cells may rely on increased glucose uptake to support their energy requirements and biosynthetic processes. Pal D et al. showed that the two telomere-binding proteins, RAP1 and POT1, in the Rap1 signaling pathway may influence RCC progression by affecting telomere stability and function [36]. Yang Y et al. discovered that MICAL1 promotes RCC cell migration by activating the downstream effector Rac1 in the Ras signaling pathway [37]. Bahar ME et al. highlighted that the aberrant activation of the Ras signaling pathway is one of the key mechanisms in the initiation and progression of RCC. Ras mutations lead to continuous activation of the MAPK pathway, triggering uncontrolled cell proliferation and resistance to apoptosis-inducing drugs, thereby affecting RCC cell proliferation and survival via regulation of the downstream RAF-MEK-ERK signaling pathway [38]. Sun Y et al. found that sphingolipid-related genes, such as SPHK1 and CERS5, play important roles in ccRCC, particularly in processes like tumor cell proliferation, migration, and survival. Most sphingolipid signaling pathway-related genes were found to have protective effects in ccRCC, suggesting they could be potential therapeutic targets for RCC [39]. Yin W et al. explored the role of purine metabolism-associated long non-coding RNAs (lncRNAs) in ccRCC, and noted that the expression of LINC01671 was associated with the survival rate of RCC patients, potentially upregulating pathways such as MAPK, NF-kappa B, mTOR, PI3K-Akt, and Wnt [40]. It is evident that the pathways such as Choline metabolism in cancer, Glycerophospholipid metabolism, Purine metabolism, Central carbon metabolism in cancer, Rap1 signaling pathway, Ras signaling pathway, and Sphingolipid signaling pathway are closely related to the development of ccRCC.Our integrated transcriptomic and metabolomic approach allows us to address these questions simultaneously. We identified Guanosine triphosphate (GTP) and L-Malic acid as central metabolites elevated in ccRCC, and their changes are mechanistically linked to the key pathways of Purine metabolism and Glycerophospholipid metabolism, respectively. This is not merely correlative. GTP directly fuels purine synthesis and provides essential energy and phosphate groups for lipid biosynthesis. L-Malic acid accumulation reflects a reprogrammed TCA cycle—often due to impaired SDH (SDHC/SDHB downregulation) or FH function—and supplies the carbon skeletons (acetyl-CoA) and reducing power (NADPH) for de novo lipogenesis, feeding into membrane phospholipid production (involving upregulated LPCAT/LPEAT).

Critically, our DEG-DAM network provides multi-omics evidence linking these metabolic shifts to specific oncogenic drivers. The MET oncogene, frequently overexpressed in ccRCC, activates the mTORC1 pathway, which transcriptionally upregulates both Purine metabolism (elevating GTP) and the lipogenic program. Concurrently, dysfunction of the mitochondrial enzymes SDHC, SDHB, and FH—common in ccRCC via mechanisms like VHL-mediated suppression or mutation—disrupts the TCA cycle, leading to oncometabolite accumulation, HIF-1α stabilization, and a metabolic state that promotes glycolysis and lipid synthesis, thereby contributing to L-malic acid accumulation and lipid droplet formation. Thus, the coordinated variations in MET and the mitochondrial genes (SDHC/SDHB/FH), as captured by our integrated analysis, converge to drive the Purine and Glycerophospholipid metabolic pathways. This elevates GTP and L-Malic acid levels, which act as both effectors and biomarkers of the lipid metabolic reprogramming that underlies ccRCC progression.

Of particular note is that metabolic alterations within tumor cells not only support their rapid proliferation but may also maintain cancer stem cell properties and contribute to therapy resistance through metabolic plasticity [41]. Targeting these metabolic vulnerabilities, especially mitochondrial metabolic dysfunction, is considered an emerging strategy to overcome treatment resistance in ccRCC [42].

In the study by di Meo NA et al., they identified 13 susceptibility genes, including VHL, MET, and BAP1, associated with renal cell carcinoma (RCC) [43]. Specifically, in our study, we found that genes such as MET, SDHC, FH, and SDHB showed significant correlations in the interaction network of differentially expressed genes (DEGs) and differentially abundant metabolites (DAMs) (Fig. 5d). Specifically, the variation in the MET gene was associated with changes in the metabolite Guanosine triphosphate. Additionally, variations in genes like SDHC, FH, and SDHB were correlated with the metabolite L-Malic acid. Based on these findings, we propose that the metabolites Guanosine triphosphate and L-Malic acid could serve as potential biomarkers for ccRCC.

The downregulation of adenosine kinase (AK) limits the phosphorylation of adenosine, which may lead to the accumulation of adenosine within the cell, subsequently affecting cellular energy metabolism and signaling [44]. As an immunosuppressive signaling molecule, high concentrations of adenosine can mediate T cell functional suppression through the A2A receptor, thereby promoting tumor immune escape [45–47]. This mechanism is particularly important in the tumor microenvironment (TME) of ccRCC, as ccRCC is one of the most immune-infiltrated tumor types [48]. Metabolite exchange and activation of specific metabolic pathways within the TME play crucial roles in regulating immune cell function and angiogenesis [49]. Cancer cells may then rely on alternative metabolic pathways, such as the Warburg effect, to compensate for their energy needs [50, 51].Consequently, the metabolic features of the TME profoundly influence disease biology and may determine patient responses to systemic therapies, including targeted therapies and immunotherapies [52, 53]. Understanding these metabolic-immune interactions is crucial for developing more effective combination strategies and identifying biomarkers predictive of therapeutic response [54].

The reprogramming of choline and glycerophospholipid metabolism, highlighted by our data, actively enables ccRCC invasion and metastasis. This is achieved by supplying essential phospholipids for rapid membrane synthesis during migration, remodeling membrane microdomains to hyperactivate promigratory signaling, and fueling the dynamic lipid droplets that provide energy and precursors for survival in distant organs. Thus, these metabolic alterations equip tumor cells with critical structural, signaling, and energetic tools for dissemination.

Phosphatidylcholine (PC) and phosphatidylethanolamine (PE) are major components of cell membranes, and the rapid proliferation of cancer cells requires an increase in membrane lipid synthesis. Cancer cells adapt to their rapid growth, invasiveness, and oxidative stress environments by altering phospholipid metabolism. Since cell proliferation depends on membrane formation, the phospholipid metabolism pathway is activated to meet these demands [55–57].

Key enzymes in phospholipid metabolism, such as AK, LPCAT, or LPEAT, could serve as potential therapeutic targets. By inhibiting these pathways, it may be possible to block tumor membrane formation and signaling. The differentially abundant metabolites, Guanosine triphosphate and L-Malic acid, along with associated genes like MET, SDHC, FH, and SDHB, may also serve as potential biomarkers for ccRCC, which could be used for diagnosis or assessing treatment efficacy.

## Conclusions

This study integrates RNA-seq and LC–MS-based metabolomics to uncover the molecular mechanisms of clear cell renal cell carcinoma (ccRCC) and identify potential therapeutic targets and biomarkers. A total of 3,237 differentially expressed genes (DEGs) and 306 differentially abundant metabolites (DAMs) were identified, and an interaction network between the two was constructed. Network analysis revealed that metabolites such as Guanosine triphosphate, Guanosine monophosphate, Glycerol-3-phosphate, and L-Malic acid were significantly enriched in tumor tissues, primarily involved in purine metabolism and glycerophospholipid metabolism pathways. These findings deepen our understanding of the molecular mechanisms underlying ccRCC progression and provide clues for the development of novel therapeutic strategies and prognostic evaluation methods.

The study also confirmed that pathways such as Choline metabolism in cancer, Glycerophospholipid metabolism, Purine metabolism, and Central carbon metabolism in cancer are closely associated with the onset of ccRCC. At the same time, genes like MET, SDHC, FH, and SDHB exhibited significant correlations in the DEG-DAM network, and their variations may contribute to tumor progression by affecting the levels of metabolites such as Guanosine triphosphate and L-Malic acid. Moreover, key enzymes in phospholipid metabolism (such as AK, LPCAT, and LPEAT) could serve as potential therapeutic targets, and inhibiting these pathways may help block tumor membrane formation and signaling. Overall, this study provides important data for elucidating the molecular mechanisms of ccRCC and may facilitate the development of novel therapeutic strategies to improve patient prognosis.

Certainly, this study has some limitations. First, the small sample size may have prevented us from capturing all potential biological variations within the ccRCC patient population. Additionally, the effects of certain metabolic pathways and genes may exhibit heterogeneity across different clinical contexts, and therefore, the applicability of the current findings to diverse populations needs further validation. To enhance the reliability and generalizability of the results, future studies should increase the sample size and consider multi-center collaborations to further confirm the roles of these key genes and metabolic pathways in ccRCC, as well as explore their potential as therapeutic targets.

## Supplementary Information

Additional file 1.

## Data Availability

The raw data of all RNA-Seq samples used in this study have been deposited in the NCBI Sequence Read archive under project ID PRJNA1204171.

## Abbreviations

ccRCC: Clear cell renal cell carcinoma
RCC: Renal cell carcinoma
DEGs: Differentially expressed genes
DAMs: Differentially accumulated metabolites
KEGG: Kyoto Encyclopedia of Genes and Genomes
GO: Gene ontology
PCA: Principal component analysis
TPM: Transcripts per million
FDR: False discovery rate
LC–MS: Liquid chromatography–mass spectrometry
RNA-seq: RNA sequencing
